# Oligofructose-Enriched Inulin Consumption Acutely Modifies Markers of Postexercise Appetite

**DOI:** 10.3390/nu15245017

**Published:** 2023-12-06

**Authors:** Courteney C. Hamilton, Marc R. Bomhof

**Affiliations:** Department of Kinesiology and Physical Education, University of Lethbridge, Lethbridge, AB T1K 3M4, Canada

**Keywords:** inulin-type fructans, prebiotic, postexercise, appetite, energy intake

## Abstract

Enhancing the effectiveness of exercise for long-term body weight management and overall health benefits may be aided through complementary dietary strategies that help to control acute postexercise energy compensation. Inulin-type fructans (ITFs) have been shown to induce satiety through the modified secretion of appetite-regulating hormones. This study investigated the acute impact of oligofructose-enriched inulin (OI) consumption after exercise on objective and subjective measures of satiety and compensatory energy intake (EI). In a randomized crossover study, following the completion of a 45 min (65–70% VO_2peak_) evening exercise session, participants (BMI: 26.9 ± 3.5 kg/m^2^, Age: 26.8 ± 6.7 yrs) received one of two beverages: (1) sweetened milk (SM) or (2) sweetened milk + 20 g OI (SM+OI). Perceived measures of hunger were reduced in SM+OI relative to SM (*p* = 0.009). Within SM+OI, but not SM, plasma concentrations of GLP-1 and PYY were increased and acyl-ghrelin reduced from pre-exercise to postexercise. EI during the ad libitum breakfast in the morning postexercise tended to be lower in SM+OI (*p* = 0.087, d = 0.31). Gastrointestinal impacts of OI were apparent with increased ratings of flatulence (*p* = 0.026, d = 0.57) in participants the morning after the exercise session. Overall, the ingestion of a single dose of OI after an exercise session appears to induce subtle reductions in appetite, although the impact of these changes on acute and prolonged EI remains unclear.

## 1. Introduction

Over the past 3 decades, there has been a steady increase in adipose-related chronic disease [1,2]. Approximately 40% of the adult population employs personal weight control efforts to lower and manage body weight [3]. With the widespread belief that exercise is highly effective for weight loss [4], exercise is one of the most utilized strategies employed by individuals to control body weight [3]. Despite the increased energy expenditure induced with exercise, exercise training has not been shown to yield anticipated weight loss [5,6]. Based on measures of fat free and fat mass after long-term exercise interventions, weight loss that is achieved represents only a fraction of exercise-induced energy expenditure, suggesting that physical activity is associated with a high degree of energy compensation [7]. 

The mechanisms underlying energy compensation are not completely understood. Evidence suggests that energy compensation may be due to either a reduction in non-exercise activity thermogenesis (NEAT) or an increase in EI following exercise. Despite the reputed transient anorectic effects associated with a bout of exercise [8], long-term exercise interventions paradoxically result in increased appetite and hunger with exercise training. Myers et al. observed that after a 12-week exercise intervention in 24 women with overweight, total energy intake and pre-meal hunger were significantly increased [6]. Based on the level of energy expenditure from the exercise sessions and anticipated weight reduction in response to the energy expenditure, energy compensation limited weight reduction to only 22% of expected values. 

With evidence of compensatory EI following exercise, there is current interest in strategies that assist with postexercise appetite management. Research has demonstrated that the consumption of prebiotics, specifically inulin-type fructans (ITF), have the potential to induce satiety through enhanced secretion of glucagon-like peptide-1 (GLP-1) and peptide YY(PYY) as well as reduced secretion of orexigenic hormone acyl-ghrelin [9]. The release of GLP-1 and PYY from enteroendocrine L-cells in the distal gut can induce satiety through activation of appetite-suppressing regions within the arcuate nucleus of the hypothalamus [10]. In contrast, acyl-ghrelin, released from gastric cells, increases neuropeptides within the hypothalamic arcuate nucleus that stimulate appetite. This action is mediated through vagal–brainstem–hypothalamic signaling or direct activation through movement of hormones across the incomplete blood–brain barrier via the median eminence [10]. ITFs are a fermentable fibre consisting of linear fructans linked with β1–2 bonds. Inulin, commonly derived from chicory, has a degree of polymerization (DP) between 2 and 60, whereas oligofructose is a shorter chain molecule with a DP < 10 [9]. As evidenced by acute increases in breath hydrogen shortly after ingestion, fermentation of inulin is initiated within hours of consumption [11]. Oligofructose, being a smaller molecule with a lower DP relative to inulin, is purported to undergo a more rapid fermentation within the GI tract [9]. The rapid fermentation of ITFs increases the production of SCFAs, which are key molecules linked to the secretion of gut-derived, satiety signals [12,13,14,15,16,17,18]. 

To date, there remains limited research investigating the acute impact of ITFs on satiety hormone secretion and energy intake during the postexercise period. Given the appetite-suppressing qualities of ITFs, there is a potential role for these dietary agents to help manage postexercise appetite. The objective of this study was to assess the acute influence of oligofructose-enriched inulin (OI) consumption after the completion of a 45 min session of moderate to vigorous exercise on appetite-regulating hormones and self-reported measures of appetite over a 12 h postexercise period in adults.

## 2. Materials and Methods

### 2.1. Participants and Ethical Approval

A total of 14 participants (8 male and 6 female) between the ages of 18–50 were recruited from the University of Lethbridge and the surrounding area. Eligibility was assessed using a screening questionnaire and interview. All participants completed the physical activity readiness questionnaire plus (PAR-Q+) to determine their ability to complete the exercise sessions [19]. If a prospective participant answered ‘yes’ to any of the questions in Section 1 and Section 2 of the PAR-Q+, participation in the study was not permitted. Recreational physical activity was assessed using a modified Godin’s leisure time exercise questionnaire (GLTEQ) [20]. To ensure that participants were accustomed to regular exercise, recreational activity was set as a minimum of 3 sessions of moderate to vigorous activity a week to the minimum recommended 150 min per week. Exclusion criteria included smoking, taking medication that could influence appetite, consumption of prebiotic or probiotic supplements, lactose intolerance, irritable bowel syndrome, and restrictive eating behaviour. Restrictive eating behaviour was assessed using the Three-Factor Eating Questionnaire Revised 18-item (TFEQ-R18) [21,22,23]. To control for appetite fluctuations throughout the menstrual cycle [24,25], female participants were tested in the early follicular phase (day 1–10), based on self-reported menstruation. All participants provided written informed consent prior to participating in the study. Ethical approval was obtained by the University of Lethbridge Human Research Committee and was conducted in accordance with the ethical principles of the 1964 Declaration of Helsinki. The results from the control session used in this study have been reported previously [26].

### 2.2. Baseline Testing

For 24 h prior to baseline testing, participants were requested to refrain from alcohol, caffeine, and moderate to vigorous exercise. Height (nearest 0.1 cm) and weight (nearest 0.1 kg) were measured using a mechanical beam scale (Heath-o-Meter Professional, McCook, IL, USA). An incremental test to exhaustion on an electromagnetically braked cycle ergometer (Velotron, QUARQ, Spearfish, SD, USA) was used to assess peak oxygen uptake (VO_2peak_). Following a 5 min warm-up at a self-selected pace, participants initiated the test at 50 W for 4 min at 80 rpm. After 4 min, the power output was increased by 25 W/min for females or 30 W/min for males. The test was terminated when participants reached volitional failure or could no longer maintain 80 rpm. Oxygen consumption was assessed through breath-by-breath analysis using a metabolic cart (Quark CPET, COSMED, Chicago, IL, USA). Heart rate was measured continuously throughout the test by a Garmin heart rate monitor (HRM-Dual, Garmin, Olathe, KS, USA). At the end of the VO_2peak_ test, VO_2peak_ was determined to be the greatest 30 s rolling average. 

### 2.3. Experimental Conditions

**Figure 1:**
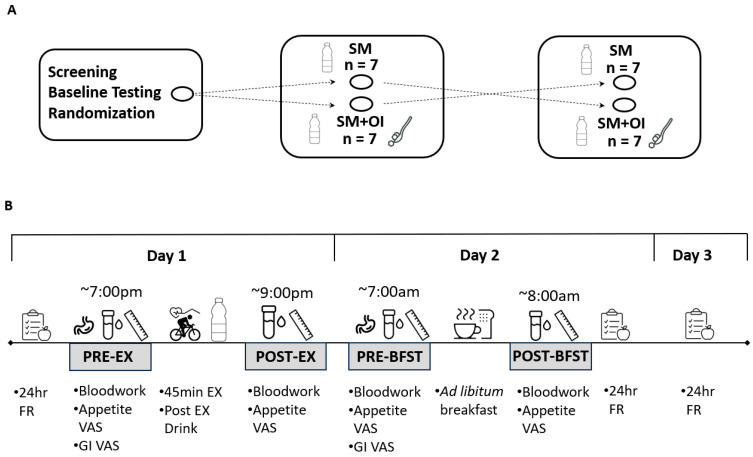
Study design. (**A**) Crossover experimental sessions, separated by 1–4 weeks. (**B**) Experimental protocol for each session. GI: Gastrointestinal; FR: food record; PRE-EX: pre-exercise; OI: oligofructose-enriched inulin; PRE-BFST: pre-breakfast; POST-BFST: post ad libitum breakfast; POST-EX; postexercise; SM: Sweetened Milk; VAS: Visual Analogue Scale.

In random order, participants completed two experimental sessions. Participants were advised to arrive at the laboratory at ~1900 h. Both sessions were performed at the same time of day. Each session included a standardized 45 min evening exercise session at 65–70% of VO_2peak_ on the cycling ergometer. VO_2_ was spot monitored during each session using the metabolic cart. Immediately after the completion of the exercise session, participants received a beverage of either (1) sweetened milk (SM) (Vanilla Vibe, Milk2Go, QC, Canada) or (2) SM with 20 g of a 50/50 mixture of oligofructose and inulin (SM+OI) (Prebiotin, Jackson GI Medical, PA, USA). For each session, the volume of SM was 10 mL/kg body weight, providing an average 495 ± 25 kcals. A 1–4 week washout period was provided between all trials. Female participants completed both experimental sessions during the early follicular phase of the menstrual cycle. All participants were asked to follow the same meal and activity patterns for the 24 h leading into the exercise sessions. Food intake was assessed using three-day food records starting the morning of the exercise session day. Participants were free to leave the laboratory after completion of the 1 h postexercise bloodwork and requested to return to the laboratory the following morning. Apart from water consumption, no food or beverage was permitted during the time period away from the laboratory. The following morning, participants reported to the laboratory around the time they would typically consume their first meal. After confirming that participants maintained the fast throughout the evening, participants received an ad libitum buffet breakfast meal consisting of instant oatmeal, orange juice, and coffee or black tea with 18% cream and/or sugar. Participants were told to consume as much or as little as they would like until they were satisfied. The meal was provided in a distraction-free office. Post-meal, all remaining food was weighed in order to calculate food consumed and EI. Venous blood samples were collected via venipuncture from the antecubital area at pre-exercise (PRE-EX), 1 h post-drink (POST-EX), and the following morning in a fasted state (PRE-BFST). Appetite and gastrointestinal perceptions were assessed using a visual analogue scale (VAS) at PRE-EX, POST-EX, PRE-BFST, and post ad libitum meal (POST-BFST). An overview of the timeline for each experimental condition is shown in Figure 1. 

### 2.4. Appetite Perceptions

Appetite perceptions were assessed using a validated 100 mm VAS [27]. Each category of appetite perceptions was anchored at 0 mm and 100 mm with opposing statements that answered each question. There were eight aspects of appetite assessed: hunger (anchored by “I am not hungry at all” and “I have never been more hungry”), satisfaction (anchored by “I am completely empty” and “I cannot eat another bite”), fullness (anchored by “Not at all full” and “Totally full”), and prospective food consumption (PFC) (anchored by “Nothing at all” and “A lot”). An additional measure of subjective appetite was calculated by taking the mean appetite rating using the 4 measures of appetite from the VAS (hunger, satisfaction, fullness, and PFC) through the composite satiety score (CSS) [28]. CSS was calculated using the equation: CSS (mm) = (satisfaction + fullness + (100 − PFC) + (100 − hunger))/4 [29]. Previous studies have utilized the CSS [28,29,30,31] as it provides a mean appetite rating of the 4 subjective measures of hunger, satisfaction, fullness, and PFC.

### 2.5. Gastrointestinal Perceptions

Gastrointestinal perceptions were assessed using a 100 mm VAS. This questionnaire assessed how gastrointestinal feelings surrounding 4 separate categories have, on average, felt over the past 12 h. Each category was anchored by opposing statements at 0 mm and 100 mm. Abdominal comfort was anchored by “Greatly improved” and “Greatly decreased”. Abdominal distention and Bloating, Flatulence or Passage of Gas, and Rumbling of your Stomach were all anchored by “No problem” and “Very Strong”.

### 2.6. Three-Day Food Diaries and Physical Activity

Three-day food diaries included the day of exercise and 2 days afterwards. Participants could track intake via an online resource (MyFitnessPal™) or by written food diaries. To ensure compliance, email and verbal reminders were implemented. Participants were given a digital food scale and instructions on completing a food diary to increase the accuracy of the diet records. Once finished participants brought their food diaries to the lab. Diaries were entered into FoodWorks 14 Software (The Nutritional Company, Long Valley, NJ, USA). EI from all three days for each condition was added together to examine the total energy intake (TEI). Weekly physical activity during the week of each experimental condition was evaluated using a modified GLTEQ.

### 2.7. Blood Collection and Analysis

Blood samples were collected into 6 mL EDTA vacutainers via venipuncture. Immediately after collection, a protease inhibitor cocktail containing dipeptidyl peptidase IV inhibitor (10 μL/mL blood; MilliporeSigma Corp., Burlington, MA, USA), sigma protease inhibitor (1 mg/mL blood; SigmaFast, MilliporeSigma Corp.) and pefabloc (1 mg/mL blood; MilliporeSigma Corp.) was added to the sample to prevent degradation of appetite-related hormones. Samples were centrifuged at 2000 rpm for 10 min at 4 °C. Plasma aliquots were stored at −80 °C for later analysis. The concentration of plasma hormones was determined using the Human PYY (Total) kits, High Sensitivity GLP-1 Active Chemiluminescent kits, and Human Ghrelin (Active) ELISA kits (MilliporeSigma Corp., Burlington, MA, USA). Inter- and intra-assay variations were 3.09 ± 3.29% and 2.39 ± 1.14% for PYY, 6.20 ± 6.71% and 6.75 ± 5.46% for GLP-1, and 2.73 ± 3.11% and 2.11 ± 1.50% for acyl-ghrelin. All samples were assayed in duplicate and samples from one participant session were analyzed in the same assay to minimize the effects of inter-assay variation.

### 2.8. Statistical Analysis

SPSS software v25.0 for Windows was used for data analysis. PD blood sample concentrations were normalized to BL values and analyzed as an absolute change in concentration. Differences in concentrations of the hormone levels, appetite perceptions, and gastrointestinal feelings across time were examined using two-way repeated-measures analysis of variance (ANOVA). When there was a condition x time interaction, pairwise comparisons were used to examine significant differences at individual time points. Within-condition differences were examined using a one-way RM-ANOVA followed by a post hoc Bonferroni analysis. Data was assessed for normality using the Shapiro–Wilk test. Cohen’s d [32] was calculated for all pairwise comparisons to examine the effect size. A small effect size is defined as <0.2, moderate effect size 0.20 ≥ 0.80, and large effect size > 0.80. Statistical significance was set at *p* < 0.05. All results are presented as mean ± standard deviation (SD). A sample size estimation of 14 was based on previously published studies using similar, postexercise research designs [33,34,35,36] and calculated in G*Power with input values of α = 5%, β = 80%, and effect size = 0.7 (moderate) using the repeated-measures ANOVA, between factor statistical test. The results from the control session used in this study have been reported previously [26]. 

## 3. Results

### 3.1. Participants

A total of 14 participants (8 male and 6 female) completed the study. Participants were 26.8 ± 6.7 years old with a BMI of 26.9 ± 3.5 kg/m^2^, height of 174.5 ± 11.1 cm, and weight of 82.5 ± 15.8 kg. Female participants were tested on day 6.6 ± 1.8 of the menstrual cycle. The average VO_2peak_ was 37.3 ± 7.5 mL O_2_·kg^−1^·min^−1^, with the average exercise intensities being 68.0 ± 4.9% and 69.1 ± 3.7% for SM and SM+OI, respectively. Average restrained eating scores were 12.2 ± 3.9.

### 3.2. Energy Intake

**Figure 2:**
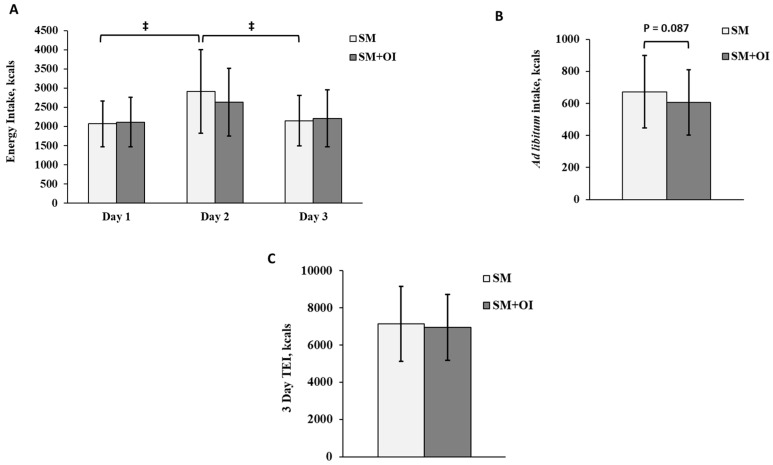
(**A**) Daily energy intake over 3 days in the SM versus SM+OI: Day 1–day of exercise; (**B**) *Ad libitum* breakfast energy intake on Day 2; (**C**) total energy intake over 3 days. ‡: significant main effect of time for SM, where *p* < 0.050. Values are means ± standard deviation, *n* = 14. SM: Sweetened milk; SM+OI: Sweetened milk + oligofructose-enriched inulin; TEI: Total energy intake.

In SM, there was a main effect of time for three-day energy intake (*p* = 0.001). On Day 2 (the day after exercise), SM consumed more energy than on Day 1 (*p* = 0.005; d = 0.96) and Day 3 (*p* = 0.021; d = 0.85) (Figure 2A). Within SM+OI, the difference between Day 1 and Day 2 was not significant (*p* = 0.099, d = 0.67). The ad libitum breakfast consumption difference between SM+OI and SM did not reach statistical significance (SM: 673 ± 227 kcal; SM+OI: 606 ± 205 kcal; *p* = 0.087; d = 0.31) (Figure 2B). Total EI remained the same between conditions (*p* = 0.667) (Figure 2C). Macronutrient distribution between the experimental conditions did not differ. 

### 3.3. Appetite-Related Hormones

**Figure 3:**
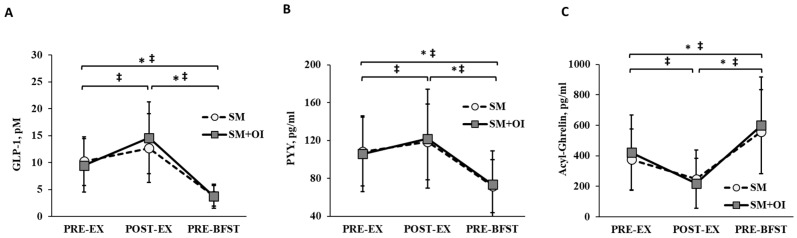
Hormone concentrations throughout experimental sessions between SM and SM+OI. (**A**) GLP-1, (**B**) PYY, and (**C**) Acyl-Ghrelin. ‡: significant within condition difference for SM where *p* < 0.050. *: significant within condition difference for SM+OI where *p* < 0.050. Values are means ± standard deviation, *n* = 14. GLP-1: glucagon-like peptide-1; PRE-EX: pre-exercise session; PRE-BFST: pre-breakfast; POST-BFST: post ad libitum breakfast; POST-EX; postexercise; PYY: peptide YY; SM: Sweetened milk; SM+OI: Sweetened milk + oligofructose-enriched inulin.

Baseline (PRE-EX) concentrations of GLP-1, PYY, and acyl-ghrelin did not differ between conditions (*p* < 0.050). There was a main effect of time for all hormones (*p* < 0.01) (Figure 3). Within SM+OI, concentrations of GLP-1 (*p* = 0.003; d = 0.88) and PYY (*p* = 0.050; d = 0.34) were greater at POST-EX compared to PRE-EX while the concentration of acyl-ghrelin (*p* = 0.003, d = 0.97) was reduced at POST-EX compared to PRE-EX. In contrast, the change between PRE-EX to POST-EX in SM was not significant for GLP-1 (*p* = 0.392, d = 0.44), PYY (*p* = 0.252, d = 0.27), and acyl-ghrelin (*p* = 0.061, d = 0.65). In both conditions, GLP-1 and PYY were reduced and acyl-ghrelin increased from PRE-EX to PRE-BFST as well as POST-EX to PRE-BFST (*p* < 0.050). 

### 3.4. Appetite Perceptions

**Figure 4:**
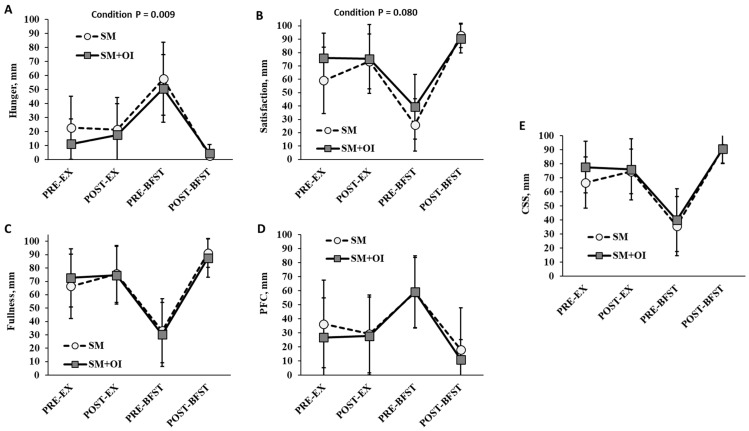
Change in perceived appetite ratings throughout experimental sessions for (**A**) Hunger, (**B**) Satisfaction, (**C**) Fullness, (**D**) PFC, and (**E**) CSS. Values are means ± standard deviation, *n* = 14. CSS, Composite Satiety Score; PFC, Prospective Food Consumption; PRE-EX: pre-exercise session; PRE-BFST: pre-breakfast; POST-BFST: post ad libitum breakfast; POST-EX; postexercise; SM: Sweetened milk; SM+OI: Sweetened milk + oligofructose-enriched inulin.

Measures of hunger, satisfaction, fullness, PFC, and CSS did not differ at PRE-EX. There was a main effect of time for subjective measures of appetite (*p* < 0.001) (Figure 4). Condition affected the hunger rating, with SM+OI demonstrating an overall reduction in hunger relative to SM (*p* = 0.009) (Figure 4A). There was a trend towards a reduction in satisfaction with SM+OI (*p* = 0.080) (Figure 4B). No differences were observed for measures of fullness (*p* = 0.916), PFC (*p* = 0.302), or CSS (*p* = 0.102) between SM and SM+OI. 

### 3.5. Gastrointestinal Perceptions

**Figure 5:**
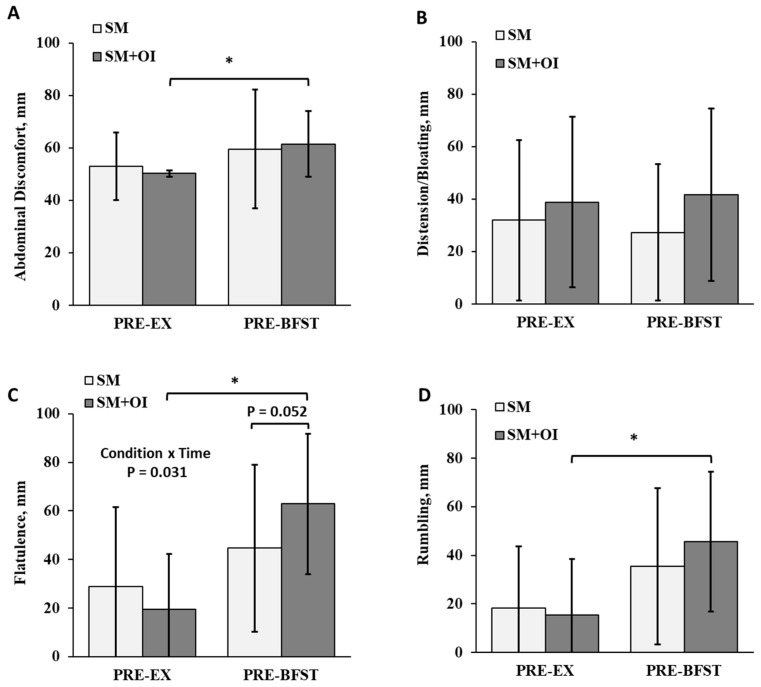
Comparison of gastrointestinal perceptions between the pre-exercise and pre-breakfast period in the SM and SM+OI conditions. (**A**) Abdominal comfort, (**B**) Distention and bloating, (**C**) Flatulence, and (**D**) Rumbling. *: significant within condition difference for SM+OI where *p* < 0.050. Values are means ± standard deviation, *n* = 14. PRE-EX: pre-exercise session; PRE-BFST: pre-breakfast; POST-BFST: post ad libitum breakfast; POST-EX; postexercise; SM: Sweetened milk; SM+OI: Sweetened milk + oligofructose-enriched inulin.

Condition and time interacted to affect measures of flatulence (*p* = 0.031) (Figure 5C). Flatulence ratings were elevated between conditions at PRE-BFST, where SM+OI reported increased flatulence compared to SM (*p* = 0.026; d = 0.57). SM+OI had within-condition changes, including an increase in abdominal discomfort (*p* = 0.005, d = 1.27) and increase in flatulence (*p* < 0.001; d = 1.67) and rumbling (*p* = 0.001; d = 1.16) from PRE-EX to PRE-BFST. Time impacted ratings of flatulence and rumbling (*p* < 0.005).

## 4. Discussion

Although exercise commonly induces a transient reduction in appetite following a session of exercise, long-term exercise is associated with increased hunger and energy compensation [7]. There is growing interest in the use of dietary strategies to manage appetite during the postexercise period [6,37]. While previous studies have noted the satiating impacts of OI, studies to date have not examined the acute impact of OI after exercise. This study evaluated the influence of OI, added to a sweetened milk beverage, on postexercise EI, appetite-regulating hormones, and subjective appetite. Participants reported reduced feelings of hunger in the SM+OI condition. Coupled with these changes, there was an elevation in GLP-1 and PYY and reduction in acyl-ghrelin from pre-exercise to postexercise in SM+OI. The fermentative action of OI within the gastrointestinal tract was evident, with increased ratings of flatulence the morning after the exercise session. Despite these noted changes in markers of appetite, there were no clear reductions in EI associated with OI intake the day following the acute exercise bout. 

Dietary fibre is one of the primary components of food associated with enhanced satiety [38,39]. In our study, we identified a hunger-reducing effect of a 20 g dose of OI over an ~15 h period following a bout of exercise. The finding of reduced hunger following acute ingestion of ITFs contrasts with previously conducted research. Prior studies examining single doses of ITF, ranging from 5–24 g, have failed to find modification of subjective appetite ratings [40,41,42,43,44,45,46]. Of note, none of the previous studies have included an exercise session prior to ingestion of the ITF. It is possible that the incorporation of the exercise session interacts with the OI ingestion to reduce subjective hunger. Although exercise commonly induces transient reductions in appetite following a session of exercise, several studies suggest that appetite may be elevated in the hours following an exercise session [47,48,49,50,51]. With an increase in postexercise hunger, there may be an enhanced sensitivity to the appetite-reducing effects of OI. Unfortunately, given that we did not include a non-exercise condition in our study, it was not possible to assess the interaction between exercise and OI on perceived hunger.

Despite a reduction in subjective hunger ratings, we did not identify a decrease in EI between the two conditions. The day following the exercise session, approximately 12 h after the bout of exercise, we identified a trend towards a 67 kcal reduction in ad libitum breakfast intake with OI. Our study was unique with the assessment of an ad libitum breakfast meal following an evening exercise session. Previously conducted research has traditionally investigated the impact of an acute dose of ITF, consumed in the morning, on ad libitum intake with a lunch meal, assessed ~4 h after fibre ingestion [40,42]. In one study conducted by Harrold et al., it was reported that a 15 g dose of inulin, consumed with a fixed load breakfast, reduced ad libitum lunch intake by 80 kcal [45]. In another study, in a group of healthy men, 16 g of inulin lowered ad libitum lunch intake by 266 kcal relative to control [46]. Furthermore, Hess et al. reported that 16 g of short-chain fructooligosaccharide reduced EI by ~370 kcal over a 24 h period. This finding, however, was only observed in females [44]. While we did not find a difference in 24 h EI between groups, assessed through food record analysis, we did observe an increase in EI from day 1 to day 2 in the SM condition that was not present in the SM+OI condition. Thus, it is possible that OI may have helped to reduce energy compensation the day following the exercise session. TEI, however, was identical between conditions over the 3-day period of study. Of note, we did not observe any differences between males and females. While short-term, acute studies have yielded mixed findings, longer-term interventions using ITFs have highlighted the effectiveness of the prebiotic supplement for inducing satiety and reducing EI [39]. In a 2 week study, Cani et al. showed that supplementation with 16 g of ITFs per day decreased total daily EI and subjective hunger [52]. Furthermore, in a 12 week study in healthy individuals with overweight or obesity, 21 g/day of oligofructose was demonstrated to reduce EI and increase weight loss [53]. Whether longer-term consumption of OI, in conjunction with exercise, would help to overcome the orexigenic effects of exercise and elicit sustained reductions in EI remains unclear and requires further examination.

Exercise sessions have been demonstrated to transiently alter appetite-regulating hormones, with increases in GLP-1 and PYY and reductions in acyl-ghrelin [54]. Similarly, ITFs have been found to increase satiety hormones and reduce orexigenic signals [55]. Here, despite no between-condition changes, we show that there was an elevation in GLP-1 and PYY and reduction in acyl-ghrelin one hour postexercise that was not evident in SM. This trend suggests that OI may elicit subtle postexercise effects within the gastrointestinal tract, leading to the altered secretion of appetite-regulating hormones. Although the mechanisms by which FOS mediates these changes remain unclear, the relatively rapid fermentation of FOS by gut microbiota, and subsequent production of SCFAs, may be in part responsible [13,56,57]. Increases in breath hydrogen, a marker of ITF fermentation, have been demonstrated to increase in a dose-dependent manner within 2 h of ITF ingestion [16,40,41,42]. In our study, with the use of oligofructose, a shorter-chain ITF, some of the OI would have fermented rapidly, potentially contributing to the subtle effects on the appetite-regulating hormones that we observed. Unfortunately, without a blood draw 2 h postexercise, we could not assess whether appetite hormones were trending up or down during this postexercise period. Several reports have indicated that the acute ingestion of inulin, consumed with a breakfast meal, is insufficient to modify GLP-1 and PYY for up to 8 h [41,42,58]. At ~12 h after the initial OI ingestion, prior to the consumption of the breakfast meal the day following exercise, our data show no differences in the concentrations of appetite hormones. The failure of ITFs to influence fasting concentrations of appetite hormones in the postexercise context is consistent within the literature. In a study conducted by Malkova et al., wherein women with overweight consumed an inulin-propionate supplement in conjunction with exercise training for a 4 week period, no changes in fasting concentrations of GLP-1 and PYY were observed [59]. Overall, the ability of ITF and associated metabolic by-products to influence appetite-regulating hormones appears to be subtle and transient.

Relative to changes in appetite, the impact of OI on gastrointestinal feelings was more pronounced. Compared to baseline measures, OI increased flatulence and led to within-condition increases in abdominal discomfort and rumbling the morning after the exercise session. Similar findings have been reported in other acute studies. In 22 healthy women, given 4 different types of fermentable fibre, bloating and flatulence increased 24 h post-consumption, with oligofructose inducing the greatest increase in GI symptoms [40]. In another study, Hess et al. reported that flatulence was the most cited GI effect associated with the consumption of a 16 g dose of short-chain fructooligosaccharide [44]. Bonnema et al. found that both 5 g and 10 g doses of inulin and oligofructose produced mild gastrointestinal bloating and flatulence within 4 h, with the symptoms tapering off after 48 h [60]. Longer-term prebiotic studies indicate that most study participants will experience some gastrointestinal side effects, ranging in severity [61,62]. It is reported that gastrointestinal symptoms associated with ITF consumption dissipate within a few days of use [63]. From our study, it is unclear if there were any interactions between exercise-induced GI effects and OI consumption. Some individuals are susceptible to exercise-induced gastrointestinal symptoms [64]. Acute ingestion of OI would likely exacerbate these symptoms, given that OI is classified as a FODMAP (fermentable oligosaccharide, disaccharide, monosaccharide, and polyols) [65]. Thus, individualized assessment of dose and tolerance would be warranted with a population utilizing postexercise ITFs as an appetite management strategy.

This study is not without limitations. The first limitation centres on the absence of a non-exercise control condition. Without this condition, it was not possible to assess interaction effects between the exercise session and ITF consumption. Secondly, this study incorporated 3-day food records to assess EI. Food records are subject to misreporting and recall bias [66], which may have affected the accuracy of the EI data collected for this study. Furthermore, given that ITFs are believed to mediate many of the impacts on appetite through gastrointestinal fermentation, our study was limited by the fact that we did not include direct measurements of prebiotic fermentation or measurement of SCFAs during the postexercise period. Measures of breath hydrogen in conjunction with the postexercise blood draws would have been beneficial. Lastly, with restrictions on exercise timing, postexercise drink volumes, fasting periods, eating time frames, and selected ad libitum breakfast food items, the conditions of this study deviate from accustomed free-living conditions. It is possible that these study restrictions influenced subjective ratings of appetite and ad libitum intake with the breakfast meal.

## 5. Conclusions

Our findings suggest that an acute dose of OI can help lower subjective measures of hunger during the postexercise period. Alongside changes in hunger, OI tended to lower subsequent EI with an ad libitum meal; however, any influence of OI on EI appears to be relatively transient. Based on these findings, postexercise OI ingestion may provide some assistance to individuals susceptible to heightened postexercise energy compensation. Presently, there remains a lack of postexercise nutritional guidelines for individuals using exercise to achieve weight loss goals. The coupling of exercise with nutrition-based strategies using prebiotics to curb postexercise appetite may serve to promote exercise adherence and help individuals with obesity and obesity-related chronic disease attain the important cardiometabolic benefits of exercise. Future studies are warranted to investigate whether repeated OI exposure after exercise limits overall energy compensation and aids in sustainable weight management with exercise training.

## Data Availability

The raw data supporting the conclusions of this article will be made available by the authors, without undue reservation.
